# *Slc20a2* is critical for maintaining a physiologic inorganic phosphate level in cerebrospinal fluid

**DOI:** 10.1007/s10048-015-0469-6

**Published:** 2015-12-12

**Authors:** Nina Jensen, Jacob Kwasi Autzen, Lene Pedersen

**Affiliations:** Department of Clinical Medicine, Aarhus University, Aarhus, Denmark; Department of Molecular Biology and Genetics, Aarhus University, C. F. Møllers Allé 3, Building 1130, 8000 Aarhus, Denmark

**Keywords:** *SLC20A2*, Inorganic phosphate, Cerebrospinal fluid, Primary familial brain calcification, Fahr’s disease, *SLC20A1*

## Abstract

Mutations in the *SLC20A2*-gene encoding the inorganic phosphate (Pi) transporter PiT2 can explain approximately 40 % of the familial cases of the rare neurodegenerative disorder primary familial brain calcification (Fahr’s disease). The disease characteristic, cerebrovascular-associated calcifications, is also present in *Slc20a2*-knockout (KO) mice. Little is known about the specific role(s) of PiT2 in the brain. Recent in vitro studies, however, suggest a role in regulation of the [Pi] in cerebrospinal fluid (CSF). We here show that *Slc20a2*-KO mice indeed have a high CSF [Pi] in agreement with a role of PiT2 in Pi export from the CSF. The implications in relation to disease mechanism are discussed.

## Introduction

Primary familial brain calcification (PFBC), formerly Fahr’s disease, is a rare autosomal dominantly inherited neurodegenerative disorder with neuropsychiatric and motor symptoms. It is characterized by calcifications in the basal ganglia and other brain regions. At least 40 % of the cases of PFBC are linked to deleterious mutations in the gene *SLC20A2*, which encodes the type III Na^+^-dependent inorganic phosphate (NaPi) symporter PiT2 [1–3], and recently, also a de novo mutation in *SLC20A2* was identified in a patient presenting with brain calcifications [4]. The mutations are predicted to result in lack of PiT2 protein or in PiT2 proteins, which are shown or predicted to be unable to transport Pi [1–17]. Both types of mutations have been suggested to result in haplo-insufficiency of Pi transport in affected cells [1]. Calcifications in PFBC have been found associated with the brain vasculature, from where they are likely to arise [18, 19]. *Slc20a2*-knockout (KO) mice present with a similar calcification phenotype [20].

A certain Pi level in the body is essential due to its buffer function and role in basic cellular processes. Pi itself is, in addition, emerging as a specific signaling molecule in mammalian cells [21]. In humans, a Pi concentration in the blood (serum phosphate) between 0.8 and 1.5 mM is considered within the normal range. Serum Pi levels above 1.5 mM (hyperphosphatemia), which are prevalent in chronic kidney disease patients, are associated with peripheral vascular calcification [22]. In vitro studies show that exposure of vascular smooth muscle cells to hyperphosphatemic conditions leads to trans-differentiation to a mineralizing cell-type [22]. In vivo, both pericytes and vascular smooth muscle cells are suggested to play active roles in peripheral vascular calcification by their trans-differentiation to mineralizing cell-types [22, 23]. Interestingly, based on studies of vascular smooth muscle cells, a key step in the Pi-induced calcification process is deregulated expression of type III NaPi symporters, which besides *SLC20A2* comprise the highly related *SLC20A1* [24]. Individuals with PFBC do, however, not show elevated serum [Pi] [1, 5, 25], and the function and role of PiT2 in relation to PFBC are not known.

There is increasing evidence that cerebrospinal fluid (CSF) composition is important in brain development and in maintenance of the health of the adult brain [26]. Recent results suggest that during sleep, the CSF follows a para-arterial pathway into the brain’s parenchyma via the so-called glymphatic system [27]. This process mixes CSF and interstitial fluid (ISF) and is suggested to lead to clearance of waste products from the brain [26, 27]. In the CSF in mammals, the [Pi] is maintained at a lower level than in the blood [28–31]. Thus, it must be expected that the ISF [Pi] is also lower than blood [Pi]. In humans, measurements on the blood and CSF taken at the same time show approximately 0.4-fold lower [Pi] in CSF [28–30]. It is not known how the CSF [Pi] is kept at a lower level than the blood [Pi].

The choroid plexus (ChP) localized at the lateral, the third, and the fourth ventricles produces most of the CSF, which has a high daily turnover rate [32]. In situ hybridization shows high expression of *Slc20a2* in mouse ChP [33]. Recently, Guerreiro and coworkers addressed the Pi transport characteristics of isolated ChP from the spiny dogfish shark, *Squalaus acanthias*, which also maintains its CSF [Pi] lower than its blood [Pi] [34]. Their results suggest that Pi is actively removed from the CSF by PiT2. Thus, analysis of the transepithelial Pi flux of ChP from the spiny dogfish shark using an Ussing chamber, identified a Na^+^-dependent Pi flux from CSF to the blood. The Pi transport characteristics were in agreement with those of PiT2 [34], e.g., it showed lithium-dependent Pi transport [7]. Only two Na^+^-dependent Pi symporters, the type III NaPi symporters PiT2 and PiT1 (encoded by *SLC20A1*), were found to be expressed in ChP from spiny dogfish sharks. PiT2 was predominantly localized to the ChP apical microvillar membranes, which faces the CSF, while PiT1 localized predominantly to the vascular endothelial cells. The localizations were confirmed on sections of rat lateral ChPs [34]. These results suggest that PiT2 plays a major role in maintaining the low [Pi] in the CSF by exporting Pi from the CSF to the blood. We hypothesized that if PiT2 is important in Pi export from the CSF, *Slc20a2*-KO mice should present with an increased CSF [Pi] compared to wild-type (WT) mice.

## Materials and methods

The breeding pairs, C57BL/6N^Tac^-*Slc20a2*^tm1a(EUCOMM)Wtsi^/Ieg (EM:05549), were obtained from the European Mouse Mutant Archive, Germany; the strain has been described previously [20]. All mice were fed with the same standard diet ad libitum. The mice were anesthetized with an initial dose of medetomidine (0.3 mg/kg), midazolam (4 mg/kg), and butorphanol (5 mg/kg), and after 15–20 min, an additional dose was given to reach a surgical plane of anesthesia. When anesthetized, the mice were positioned in a stereotaxic frame, and CSF was drawn from the cisterna magna using a glass capillary. The mice were sacrificed, and the blood was immediately sampled by cardiac puncture. The [Pi] in CSF and serum was determined using a malachite green-based assay as described previously [35]. Data were analyzed by a Welch’s *t* test using R version 3.2.2 [36]. Mean values were considered different when *P* < 0.05.

## Results

**Fig. 1:**
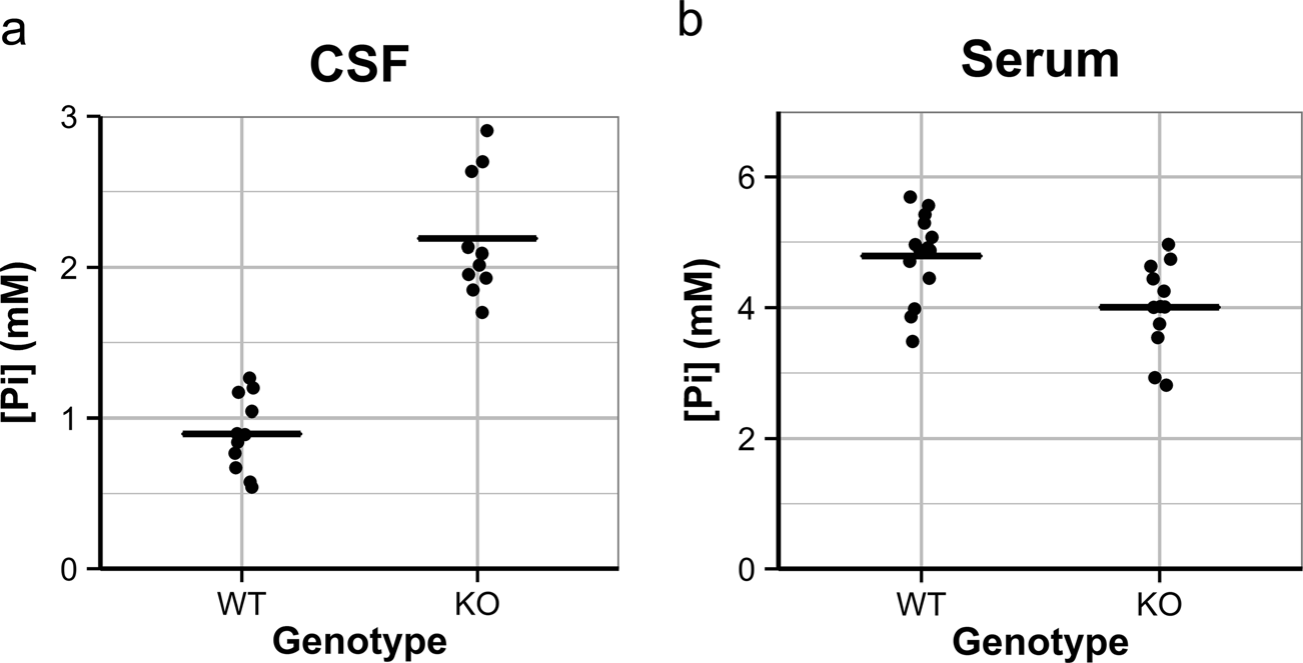
[Pi] in serum and CSF from 3-week-old WT and *Slc20a2*-KO mice. **a** CSF sampled from 11 WT (8 females, 3 males) and 10 KO (8 females, 2 males). **b** Serum sampled from 14 WT (10 females, 4 males) and 12 KO (9 females, 3 males). KO compared to WT: CSF [Pi] (*p* < 0.000001) and serum [Pi] (*p* < 0.01)

To address whether *Slc20a2*-KO mice have an elevated [Pi] in the CSF, we measured the [Pi] in CSF and blood drawn from 3-week-old *Slc20a2*-KO mice and WT litter mates. In average, the WT mice showed a serum [Pi] of 4.79 ± 0.65 mM (standard deviation) and a CSF [Pi] of 0.90 ± 0.25 mM (Fig. 1). The average CSF [Pi]/serum [Pi] ratio was 0.19 (range 0.11–0.32) (Table 1). To our knowledge, the [Pi] of CSF of mice has not previously been published; for comparison, the corresponding ratio reported in 3-week-old rats is approximately 0.17 [31].

**Table 1:** [Pi] in serum and CSF from 3-week-old WT and *Slc20a2*-KO mice

Genotype	Sex	[Pi]^a^ serum	[Pi] CSF	[Pi] CSF/[Pi] serum
WT	Female	5.07	1.20	0.24
WT	Female	5.69	ND	–
WT	Female	5.56	1.04	0.19
WT	Female	4.71	0.54	0.11
WT	Female	3.48	0.84	0.24
WT	Female	4.96	0.67	0.14
WT	Female	4.91	0.77	0.16
WT	Female	5.29	ND	–
WT	Female	3.86	0.58	0.15
WT	Female	4.87	0.89	0.18
WT	Male	4.87	1.17	0.24
WT	Male	3.98	1.26	0.32
WT	Male	5.42	0.90	0.17
WT	Male	4.45	ND	–
KO	Female	4.63	ND	–
KO	Female	4.25	1.93	0.45
KO	Female	4.96	ND	–
KO	Female	4.02	1.70	0.42
KO	Female	4.74	1.95	0.41
KO	Female	2.81	2.09	0.74
KO	Female	ND	2.63	–
KO	Female	4.01	2.13	0.53
KO	Female	2.92	2.90	0.99
KO	Female	3.75	1.85	0.49
KO	Male	4.44	2.70	0.61
KO	Male	4.01	ND	–
KO	Male	3.54	2.01	0.57
Average for WT mice with CSF sample		4.70	0.90	0.19
Average for all WT mice		4.79	–	–
Average for KO mice with CSF sample		3.83	2.19	0.57
Average for all KO mice		4.01	–	–

*ND* not done

^a^[Pi] in millimolar

The average serum [Pi] of the *Slc20a2*-KO mice was 4.01 ± 0.67 mM, which was 0.84-fold lower than the average serum [Pi] in WT mice (*p* < 0.01) (Fig. 1). However, the average CSF [Pi] of the *Slc20a2*-KO mice was 2.19 ± 0.41 mM, i.e., 2.4-fold higher than the average CSF [Pi] at 0.90 mM of WT mice (*p* < 0.000001) (Fig. 1). Correspondingly, the average CSF [Pi]/serum [Pi] ratio of the *Slc20a2*-KO mice was 0.57 (range 0.41–0.99) (Table 1). Thus, *Slc20a2*-KO mice were unable to sustain their CSF [Pi] at the same low level as the WT mice.

## Discussion

It is now well-established that deleterious mutations in the gene *SLC20A2* are linked to PFBC [1–17], which is characterized by cerebrovascular-associated calcifications [18, 19]. A similar calcification phenotype is present in *Slc20a2*-KO mice [20]. The disease mechanism is unknown, but recent in vitro results [34] suggested a role of PiT2 in maintaining the low CSF [Pi] observed in healthy individuals. The here observed inability of *Slc20a2*-KO mice to sustain the same low CSF [Pi] as found in WT mice is in agreement with a role of PiT2 as an exporter of Pi from the CSF. The elevated CSF [Pi] likely also leads to an elevation of ISF [Pi]. With reference to hyperphosphatemia, we suggest an introduction of the term CSF hyperphosphate to describe Pi levels in the CSF above the highest concentration occurring in the non-pathologic situation.

To our knowledge, the CSF [Pi] in individual carriers of *SLC20A2*-associated PFBC has not been investigated, and it remains to be seen whether they also present with CSF hyperphosphate. The here presented new insight in the role of PiT2 in maintaining CSF normophosphate in mice, however, points at a potential mechanism, or contributing mechanism, behind the cerebral vessel disease present in *SLC20A2*-associated PFBC and sporadic cases, i.e., CSF hyperphosphate. Specifically, pericytes/vascular smooth muscle cells in the brain might react in a similar manner to CSF hyperphosphate, as the peripheral vascular cells are suggested to react to hyperphosphatemia [22, 23], i.e., by shifting phenotype and being actively involved in the calcification of the blood vessels. This hypothesis does not exclude that impaired PiT2 function in specific cell types in the brain outside the ChP, e.g., in the pericytes/vascular smooth muscle cells, also directly contributes to the disease development. The presented results, however, provide the first insight into PiT2’s function in normal brain physiology in an animal model presenting with a similar calcification phenotype as found in PFBC.

Recently, mutations in another gene encoding a protein associated with cellular Pi homeostasis, *XPR1*, were also found associated with PFBC [37]. However, while *SLC20A2* encodes an importer of Pi into cells [38, 39], *XPR1* encodes a protein exporting Pi out of cells [40]. XPR1 proteins harboring damaging mutations associated with PFBC showed severely reduced membrane localization and/or impaired ability to export Pi out of cells to various degrees [37]. Interestingly, in situ hybridization shows expression of *Xpr1* in mouse ChP [33]. Albeit the exact position of XPR1 in the ChP is not clear, it is tempting to speculate that it could be positioned in the basolateral membrane of the choroidal ependymal cells and be involved in Pi export from the cells to the blood side. Accordingly, impaired XPR1 transport could lead to increased intracellular Pi accumulation. Results on non-polarized mammalian cells in culture suggest that increased intracellular [Pi] might downregulate PiT2 expression in a cell-line specific manner [38, 41]. Thus, a potential increase in intracellular [Pi], due to impaired XPR1 function, might result in downregulation of PiT2-mediated apical Pi transport from the CSF into the ependymal cells. If the hypothesis is correct, damaging *XPR1* mutations could potentially also result in CSF hyperphosphate.
